# Instillation of Six Different Ultrafine Carbon Particles Indicates a Surface Area Threshold Dose for Acute Lung Inflammation in Mice

**DOI:** 10.1289/ehp.8266

**Published:** 2005-09-22

**Authors:** Tobias Stoeger, Claudia Reinhard, Shinji Takenaka, Andreas Schroeppel, Erwin Karg, Baerbel Ritter, Joachim Heyder, Holger Schulz

**Affiliations:** GSF-National Research Center for Environment and Health, Institute of Inhalation Biology, Muenchen-Neuherberg, Germany

**Keywords:** air pollution, dose–response relation, nanoparticles, particle toxicology, particulate matter, specific surface area, ultrafine particles

## Abstract

Increased levels of particulate air pollution are associated with increased respiratory and cardiovascular mortality and morbidity. Some epidemiologic and toxicologic research suggests ultrafine particles (UFPs) (< 100 nm) to be more harmful per unit mass than larger particles. Our study was aimed at a quantitative comparison of acute adverse effects of different types of carbonaceous UFPs at a dose range that causes a moderate inflammatory response in lungs. We used six different particle types (primary particle size 10–50 nm, specific surface area 30–800 m^2^/g, and organic content 1–20%): PrintexG, Printex90, flame soot particles with different organic content (SootL, SootH), spark-generated ultrafine carbon particles (ufCP), and the reference diesel exhaust particles (DEP) SRM1650a. Mice were instilled with 5, 20, and 50 μg of each particle type, and bronchoalveolar lavage was analyzed 24 hr after instillation for inflammatory cells and the level of proinflammatory cytokines. At respective mass-doses, particle-caused detrimental effects ranked in the following order: ufCP > SootL ≥ SootH > Printex90 > PrintexG > DEP. Relating the inflammatory effects to the particle characteristics—organic content, primary particle size, or specific surface area—demonstrates the most obvious dose response for particle surface area. Our study suggests that the surface area measurement developed by Brunauer, Emmett, and Teller is a valuable reference unit for the assessment of causative health effects for carbonaceous UFPs. Additionally, we demonstrated the existence of a threshold for the particle surface area at an instilled dose of approximately 20 cm^2^, below which no acute proinflammatory responses could be detected in mice.

Numerous epidemiologic studies have demonstrated an association between elevated levels of ambient particles and morbidity or mortality. High levels of particulate matter (PM) seem to be especially harmful to susceptible sub-populations such as the elderly, patients with preexisting cardiopulmonary diseases, and diabetics (Bateson and Schwartz 2001). Ambient fine-mode PM (< 2.5 μm particle diameter, PM_2.5_) consists mainly of anthropogenic, carbonaceous particles derived from combustion processes. In urban air, fine and ultrafine particles (< 100 nm) are most numerous among all particles and represent the highest surface area per mass. This surface can carry large amounts of adsorbed or condensed toxic air pollutants such as organic compounds and transition metals (Oberdorster 2001). Although the mass of ultrafine particles (UFPs) is at ambient background levels < 2 μg/m^3^ (Hughes et al. 1998), it can increase severalfold at locations with high volumes of traffic (Zhu et al. 2002) or during high-pollution episodes, where number concentrations > 100,000 particles/cm^3^ have been measured (Brand et al. 1992; Oberdorster et al. 2000). Ultrafine components of particulate air pollution may result in local and systemic oxidative stress, which produces lung inflammation, as well as systemic effects that result in mortality from cerebrovascular disease in susceptible individuals (MacNee and Donaldson 2000).

Once inhaled, particles < 100 nm in particular are efficiently deposited in the respiratory tract (fractional deposition up to 70%), translocate efficiently into the interstitium, and largely evade macrophage clearance (Daigle et al. 2003; Ferin et al. 1992; Hahn et al. 1977). Accordingly, several epidemiologic studies have found that adverse respiratory effects are correlated to the number concentration of UFPs (Utell and Frampton 2000; Wichmann et al. 2000). Furthermore, some epidemiologic and toxicologic research suggests that UFPs are more harmful per unit mass than larger particles (Oberdorster 2001; Peters et al. 1997; Seaton et al. 1995). For example, after the German unification, efforts toward the improvement of air quality substantially decreased the mass concentrations for many pollutants but failed to cut down the number concentrations (i.e., the fraction of UFPs < 30 nm). In Erfurt, Germany, number concentrations of these particles increased by > 100% between 1991 and 1998 (Ebelt et al. 2001; Kreyling et al. 2003). In response to legal regulations, modern engines emit much lower particulate mass concentrations, but at the expense of increasing number concentrations within the sub-100 nm particle fraction.

A key issue concerning the toxicology of PM is the question whether threshold concentrations exist, below which air pollution has no effect on human health. Exposure–response curves from multiple locations in the United States and Spain have been analyzed (Schwartz and Zanobetti 2000; Schwartz et al. 2002). The results suggested a linear relationship between daily PM_10_ (PM < 10 μm in diameter) and PM_2.5_ and the number of deaths down to lowest observed exposure concentrations of 2 μg/m^3^, which gives rise to the no-threshold hypothesis. However, some animal studies suggest a threshold level for PM and also point out that upon transition from the fine to ultrafine particle size range, particle number or surface area, not particle mass, may be more appropriate to characterize adverse effects of PM (Brown et al. 2000; Oberdorster 2002; Renwick et al. 2004; Tran et al. 2000). However, no corresponding data are as yet available to substantiate these findings for the UFP size range only. Therefore, in our study we address the questions of *a*) which of the easily accessible parameters often applied in epidemiologic and toxicologic studies—particle size, surface area, or organic content (OC)—is most appropriate to characterize the inflammatory potency within the ultrafine size range; and *b*) whether we would be able to detect a threshold level for these particles under controlled experimental settings.

We investigated acute adverse effects of six types of carbonaceous UFPs by intratracheal instillation in healthy mice: two commercially available pigments (PrintexG and Printex90); two laboratory-made flame soot particles with different OC [SootL (low) and SootH (high)]; spark-generated ultrafine carbon particles (ufCP); the standardized reference material (SRM) diesel exhaust particles (DEP) SRM1650a. These particles cover a size range of 10–50 nm. The specific surface area varies between 30 and 800 m^2^/g, and the OC is between 1 and 20%. To characterize the acute inflammatory events in the lung caused by the particles, mice were killed 24 hr after instillation, and bronchoalveolar lavage (BAL) was performed immediately postmortem.

Relating the inflammatory dose effect in the lungs to particle characteristics (OC, primary particle size, and specific surface area) revealed an obvious correlation for particle surface area in particular. This suggests that specific particle surface area may be a valuable reference parameter for the characterization of detrimental health effects caused by inhaled carbonaceous UFPs. Additionally, our data point to the existence of a threshold below which no acute proinflammatory response in the lung could be found to low doses of instilled particles.

## Materials and Methods

### Particles.

We obtained standard reference material DEP from the National Institute of Standards and Technology (Gaithersburg, MD, USA). Pigment blacks PrintexG and Printex90 were obtained from Degussa (Frankfurt, Germany). SootH, SootL, and ufCP were generated and filter-sampled in our laboratory in an lipopolysaccharide-free environment. SootH and SootL aerosols were produced by means of a diffusion flame from a mixture of propane and air (CAST, Matter Engineering AG, Wohlen, Switzerland) at equal settings to ensure particle agglomerates with high (SootH) or low OC (SootL) at the same size distribution (Schroeppel et al. 2003). The ufCP particles were generated by spark discharge. The methodology of generation and a detailed description of physical particle properties have been published by Roth et al. (2004).

### Measurement of physical/chemical particle properties.

We assessed primary particle size by transmission electron microscopy (TEM) of particles resuspended in water. The six particle suspensions were prepared in the same way as for the respective instillation procedures and were immediately mounted on the TEM-grids as described by Yokota and Fujimori (1992). At least 50 particle diameters were analyzed per particle category. Specific particle surface area was assessed by the Brunauer, Emmett, and Teller (BET) method at the Technical University of Munich, Institute of Technical Chemistry. The BET method calculates the specific surface area from the quantity of a particular gas being absorbed in multi-molecular layers on the surface of the respective particle (Brunauer et al. 1938). The results were consistent with the corresponding datasheets of PrintexG and Printex90. The OC of the applied particles was measured by a thermooptical analysis technique at Clarkson University, New York, Department of Chemical Engineering, according to the National Institute for Occupational Safety and Health (NIOSH) 5040 method (described by Cassinelli and O’Connor 1998) or taken from the manufacturer’s data sheet (DEP). The range of primary particle diameter, together with organic mass content and specific surface area (BET), is shown in Table 1.

### Animals.

We received female BALB/cJ mice (Jackson Laboratory, Bar Harbor, ME, USA) at 8 weeks of age. The animals were kept in isolated ventilated cages (IVC-Racks; BioZone, Margate, UK) supplied with filtered air, in a 12-hr light/12-hr dark cycle. Specific pathogen-free hygienic status was approved by a health certificate according to the Federation of European Laboratory Animal Science Associations guidelines (Nicklas et al. 2002). Food and water were available *ad libitum*. Animals were 10–12 weeks of age with body weights between 19.6 and 23.1 g during the study. Each of the 22 experimental groups consisted of eight animals. Twenty groups were exposed to particles, and two groups served as control and sham exposed. Mice were anesthetized by intraperitoneal injection of a mixture of xylazine (4.1 mg/kg body weight) and ketamine (188.3 mg/kg body weight). The animals were then intubated by a nonsurgical technique (Brown et al. 1999). Using a bulb-headed cannula inserted 10 mm into the trachea, a suspension containing 5, 20, or 50 μg particles, respectively, in 50 μL pyrogene-free distilled water was instilled, followed by 100 μL air. (For supplementary measurements, we used 0.5 and 2 μg ufCP.) The suspension of poorly soluble particles was sonicated on ice for 1 min prior to instillation, using a SonoPlus HD70 (Bachofer, Berlin, Germany) at a moderate energy of 20 W. We favor the use of distilled water for suspension of particles because the salt content of phosphate-buffered saline (PBS) causes rapid particle aggregation comparable to the “salting-out” effect (Shaw 1992) and thus eliminates consistent instillation conditions. In our experience, the instillation of 50 μL distilled water did not cause any measurable stress effects such as the expression of heat shock protein hsp70/hsp1a (data not shown). Control animals were not instilled, and sham animals received 50 μL pure distilled water. Animals were treated humanely and with regard for alleviation of suffering; experimental protocols were reviewed and approved by the Bavarian Animal Research Authority (approval no. 211-2531-108/99).

### Bronchoalveolar lavage and analysis.

Twenty-four hours after instillation, mice were anesthetized by intraperitoneal injection of a mixture of xylazine and ketamine and killed by exsanguination. We performed BAL 24 hr after instillation. BAL was performed by cannulating the trachea and infusing the lungs 10 times with 1.0 mL PBS without calcium and magnesium, as described previously (Stoeger et al. 2004). The BAL fluids (BALF) from lavages 1 and 2 and from lavages 3–10 were pooled and centrifuged (425 × *g*, 20 min at room temperature). The cell-free supernatant from lavages 1 and 2 were pooled and used for biochemical measurements such as lactate dehydrogenase (LDH), total protein, and cytokine concentration. The cell pellet was resuspended in 1 mL RPMI 1640 medium (BioChrome, Berlin, Germany) and supplemented with 10% fetal calf serum (Seromed, Berlin, Germany); the number of living cells was determined by the trypan blue exclusion method. We performed cell differentials on the cytocentrifuge preparations (May-Grünwald-Giemsa staining; 2 × 200 cells counted). We used the number of polymorphonuclear leukocytes (PMNs) as a marker of inflammation. LDH activity was assayed spectrophotometrically by monitoring the reduction of NAD+ at 366 nm in the presence of lactate. Total protein content was determined spectrophotometrically at 620 nm, applying the Bio-Rad Protein Assay Dye Reagent (no. 500-0006; BioRad, Munich, Germany). We analyzed 50 μl BALF/mouse to assess lavage cytokine concentrations for tumor necrosis factor-α (TNF-α), interleukin 1β (IL-1β), macrophage inflammatory protein 2 (MIP2), and cytokine-induced neutrophil chemoattractant (KC), using commercial enzyme-linked immunosorbent assays (R&D Systems, Wiesbaden, Germany).

### Statistical analyses.

All values are reported as the mean ± SE of eight animals. We used analysis of variance (ANOVA), as calculated by the commercial statistical package Statgraphics (STSC Inc., Rockville, MD, USA), to establish the statistical significance of differences between the experimental groups. We applied the Tukey honestly significant difference procedure to evaluate the significant differences between the 18 particle groups (6 particles, with 3 doses each) and the sham control group. Differences were considered significant at *p* < 0.05. Single regression models were applied to relate particle properties to the measured effect levels, and a multiple linear regression model was used to assesss simultaneously the effect of particle surface area and OC in relation to the inflammatory response. We used response values of individual animals for correlation analysis.

## Results

### Grade of inflammatory response to carbon is strongly dose- and particle-dependent.

In all but two experimental groups, protein concentration in BALF remained unchanged. Only SootL and ufCP at 50 μg significantly elevated total protein levels > 300 μg/mL, compared with a level of about 200 μg/mL in control and water-instilled animals (data not shown). Even at the highest doses, none of the six investigated particle types significantly altered LDH levels within 24 hr after instillation (data not shown). Similarly, the number of nonvital leukocytes retrieved by BAL remained unaffected in all groups (data not shown). Taken together, the latter findings point to the absence of cytotoxic short-term effects of the analyzed particles up to the dose of 50 μg/mouse.

**Figure 1:**
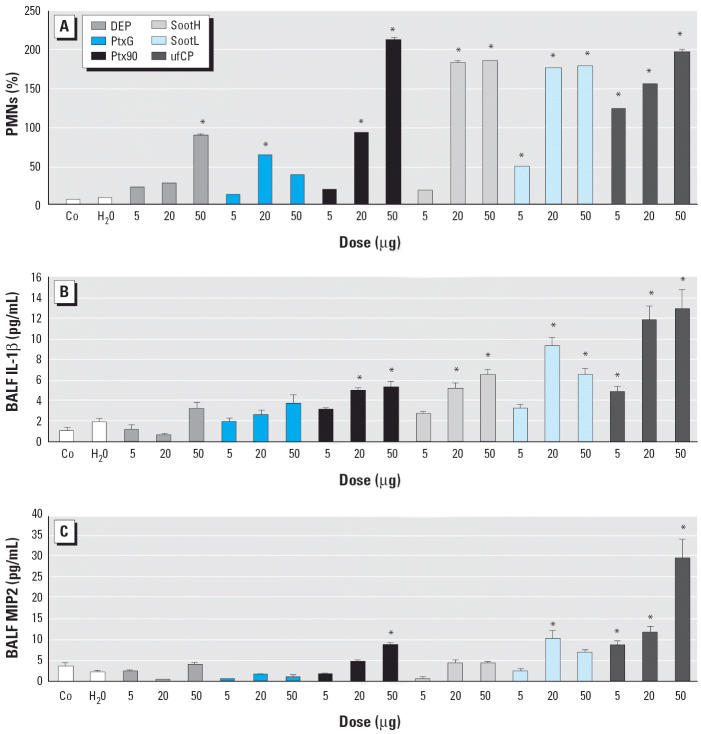
Particle-dependent elevation of PMNs (*A*), IL-1β (*B*), and MIP2 (*C*) in BAL from noninstilled (Co) and water-instilled (H_2_O) control mice and mice instilled with water, DEP, PrintexG (PtxG), Printex90 (Ptx90), SootH, SootL, and spark-generated ufCP of increasing inflammatory potency. Results are expressed as a function of gravimetric dose (5, 10, and 50 μg). Bars represent mean ± SE of eight animals. *Significantly elevated compared with Co and H_2_O controls (*p* < 0.05).

The total number of retrieved BAL leukocytes remained unchanged across the different doses and particles studied. Thus, we took the percentage of BAL neutrophils as a cellular marker to quantify early lung inflammation. As shown in Figure 1A, each particle type caused a dose-dependent inflammatory response, and all particles evoked a significant PMN influx into the alveolar space at a dose of 50 μg/mouse. Notably, instillation of water produced no altered PMN level compared with untreated control animals. At the lowest dose (5 μg/mouse), only SootL and ufCP caused significant accumulation of neutrophils in BALF. Among the six types of carbonaceous UFPs, ufCP gave the most marked inflammatory response, leading to BALF PMN contents of about 60% at a dose of 50 μg and of > 20% at 5 μg. However, DEP and PrintexG failed to cause a significant PMN influx, even at the intermediate dose of 20 μg.

To characterize the degree of inflammatory changes in the lung at a molecular level, we analyzed the amount of various pro-inflammatory cytokines released into the alveolar space: IL1-β and TNF-α, two cytokines commonly produced by activated monocytes, and MIP2 and KC, two potent neutrophil attractants that represent the murine functional homologs to human IL-8. Comparable with the conditions for PMN influx, the instillation of water did not alter cytokine concentrations in BALF, and the instillation of ufCP particles generated the highest cytokine levels at each dose. BALF concentrations of IL-1β are shown in Figure 1B. IL-1β content showed a significant increase at all ufCP doses and at higher doses of SootL, SootH, and Printex90 particles. PrintexG and DEP generally failed to increase IL-1β cytokine levels significantly. Only the instillation of ufCP significantly elevated TNF-α concentration in BAL at all three doses (sham, 2.5 ± 0.8 pg/mL; 5 μg UfCP, 7.1 ± 1.0 pg/mL; 20 μg UfCP, 7.4 ± 1.2 pg/mL; 50 μg UfCP, 15.7 ± 0.9 pg/mL). BALF concentrations of MIP2 are shown in Figure 1C. MIP2 levels were significantly increased in a dose-related manner by all doses of instilled ufCP and by the highest Printex90 dose. PrintexG and DEP did not alter MIP2 levels at this time point. SootL at 20 μg raised MIP2 concentrations significantly, whereas SootH did not. KC concentrations resembled the pattern of MIP2, reaching the highest levels of 243 ± 49 pg/mL after instillation of 50 μg ufCP (data not shown). In general, cytokine concentrations reflected a graduated dose response analogous to that observed for PMN influx.

### The inflammatory response level is most strongly related to specific particle surface area and points to a threshold for inflammation onset.

**Figure 2:**
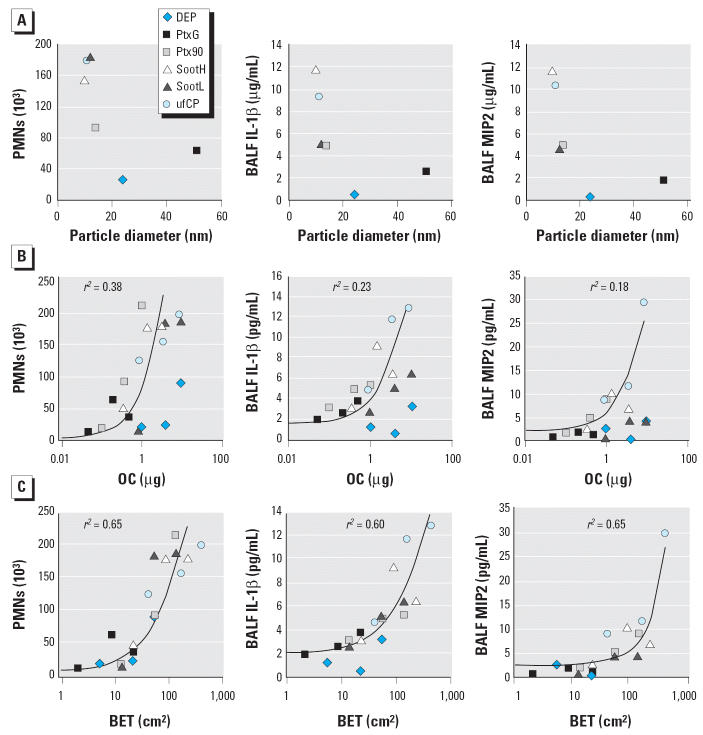
PMN cell numbers and IL-1β and MIP2 concentrations are plotted as a function of different characteristics of the instilled UFPs: (*A*) primary particle diameter (for an exemplary dose of 20 μg); (*B*) OC mass; and (*C*) particle surface area (BET). Each symbol represents mean values of eight animals. A regression analysis revealed a strong logarithmic relation for the surface area (*r*^2^ = 0.65) and a weaker relation for the organic contribution (*r*^2^ = 0.38).

Primary particle size, OC, and particle surface area at a given dose were related to end points of lung inflammation and inflammatory cell activation. In Figure 2A, the inverse correlation between primary particle size and inflammatory response, reflected by the PMN influx and MIP2 or IL-1β concentrations in BALF, is presented for the exemplary dose of 20 μg. Smaller particles obviously have a higher proinflammatory potency than larger specimens at comparable doses. As shown in Figure 2B, we found no strong correlation between OC of particles and PMN influx, MIP2, or IL-1β levels. DEP, which contained the highest fraction of organics, tended to be a less potent effector of inflammation than particles with the least OC (Printex90). Arranging the effects values of all single animals for a logarithmic equation resulted in a coefficient of determination of *r*^2^ = 0.38. In contrast, a relatively strong correlation (*r*^2^ = 0.65) became evident when the inflammation response was related to particle specific surface area for all six types of instilled particles (Figure 2C). The correlation between BET surface area and inflammatory response holds true for PMN influx and for MIP2 and IL-1β levels, as well as for KC (data not shown). To simultaneously assess the effect of surface area and OC in relation to the inflammatory response, we applied a multiple linear regression model. As shown in Table 2, there is a significant association between the inflammation end point (PMN) and the particle properties OC on one hand, and BET on the other. However, the association was highest for surface area (*p* < 0.001).

**Figure 3:**
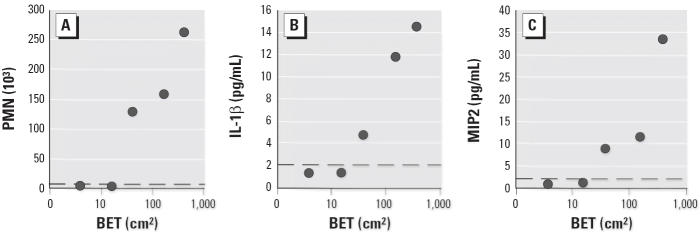
Threshold for inflammatory response for ufCP shown as the correlation between particle surface area and PMN cell numbers (*A*) or IL-1β (*B*) or MIP2 (*C*) concentrations. Two additional instilled doses of 0.5 and 2 μg, together with 5, 20, and 50 μg ufCP represent BET surface areas of 0.4, 16, 40, 161, and 404 cm^2^, respectively. The dashed line indicates baseline levels of control animals; each circle represents the mean of eight animals.

The particle surface area from 5 to 40 cm^2^ shown in Figure 2C suggests the existence of a dose–response threshold, below which no significant inflammatory reaction was detected. Taking BAL PMN influx as an end point of the inflammatory effect, the investigation for the respective no observed adverse effect level (NOAEL) points to the 5-μg Printex90 instillation, with a surface area of 13.6 cm^2^, and for the lowest observed adverse effect level (LOAEL) to a surface area of 22.1 cm^2^, represented by the 5-μg SootL dose. Consistently, the dose–effect curves for all inflammatory end points, most evidently for BALF cytokine levels, feature a point that marks the onset of a measurable inflammation response. Below this threshold, no particle-related increase in BAL PMN counts or cytokine concentrations was measurable. To confirm this threshold phenomenon, we instilled two additional doses of our most potent particle specimen, the ufCPs, at quantities < 5 μg. In concordance with comparatively low surface burdens of the other particles, BET surface doses of 4 cm^2^ and 16 cm^2^, representing ufCP particle quantities of 0.5 and 2 μg, did not induce a significant PMN influx or alter basal cytokine concentrations in BALF within 24 hr (Figure 3). Implementing these latter results into the NOAEL inspection above results in an intercept from 16 to 22 cm^2^, where a surface area response threshold should be expected.

## Discussion

The goals of our study were *a*) to investigate adverse effects of ultrafine laboratory-generated surrogates and a standardized exhaust PM at a dose range that causes moderate inflammatory responses in the lung, and *b*) to assess which of the easily accessible parameters often applied in epidemiologic and toxicologic studies—particle size, surface area, or OC—is most suitable to characterize the inflammatory potency within the UFP size range. None of the instilled materials at any dose caused an increase in LDH concentration or the number of dead cells in BALF. This finding indicates the absence of acute tissue damage within 24 hr, even at the highest doses for all six types of carbonaceous nanoparticles, and is an indication for moderate doses of particles. Nevertheless, neutrophil recruitment of up to 60% was induced at the highest doses. This response is within the range of those described by others using various types of particles (Chang et al. 2005; Dick et al. 2003 Gilmour et al. 2004; Lambert et al. 2003; Yanagisawa et al. 2003).

Several *in vivo* studies have shown that, upon transition from the fine to ultrafine size range, diameter and surface area of inhaled or instilled particles are important factors influencing the inflammatory response in the lungs (Brown et al. 2000; Oberdorster 2002; Renwick et al. 2004; Tran et al. 2000). Here we show for the first time that even within the ultrafine size range, the comparison of different nanosize (10–50 nm) carbon particles yields similar results with respect to their proinflammatory potency. In this study, among chemically identical pigments, the 14-nm Printex90 particles proved to be at least 2.5-fold more dose effective than the 50-nm PrintexG particles. Moreover, this experimental survey is the first attempting to relate the organic mass contribution of UFPs to the resultant inflammatory effect levels.

Coefficients of determination from regression analysis and subsequent multiple linear regression modeling (Table 2) suggest a less significant contribution to PMN recruitment and proinflammatory cytokine release for OC compared with particle surface area. This finding contrasts with recent investigations, which related the induction of oxidative stress to the OC—in particular, to the content of poly-aromatic hydrocarbons (PAHs)—of DEP (Li et al. 2002). For the applied flame soot particles, the mass fraction of the 16 PAHs listed by the U.S. Environmental Protection Agency is 1% for SootH and only 0.02% for SootL (Schroeppel et al. 2003); the mass fraction for DEP (SRM1650a) is 0.03%. In our study, however, at all doses investigated, SootL proved to be at least as potent as SootH and was always more potent than DEP. These results suggest that the PAH content of particles is not the major organic component driving the inflammatory response in our study. Particle-induced oxidative stress is expected to stimulate the production and release of inflammatory mediators (Donaldson et al. 2003). Beck-Speier et al. (2005) demonstrated a high oxidative potential for ufCP compared with Printex90 or DEP (SRM1650a). Consistently, an *in vitro* assay proved that ufCP particles are by far more potent in inducing oxidative stress in alveolar macrophages than Printex90 (Beck-Speier et al. 2001). In agreement with that, our *in vivo* study demonstrated that ufCP particles are the most potent inducers of acute proinflammatory responses, suggesting that oxidative stress is a main effector. Shi et al. (2003) showed that the formation of radical species also holds true for the ultrafine fraction of ambient particles. Using Printex90 and ultrafine metallic particles (cobalt, nickel, and titanium), Dick et al. showed that surface reactivity—in particular, the existence of reactive compounds on a particle surface—can substantially modify adverse effects (Dick et al. 2003). This is in agreement with our observation that surface area is most appropriate to assess the dose to which the lung is exposed. Therefore, our *in vivo* data are well in line with those published to date and support the view that the surface reactivity of carbonaceous UFPs triggers oxidative stress and inflammatory downstream effects.

Epidemiologists have observed an apparent relation between PM and adverse health effects. This raises the critical question of which easily accessible parameter most appropriately describes the exposure dose. Particle mass concentration is commonly used, although some researchers have noted that this parameter may not be appropriate because the contribution of the hazardous ultrafine-size particles to overall ambient particle mass is almost negligible (Kreyling et al. 2003). Therefore, particle number concentration, which gives high weight to the fraction of UFPs, has been applied recently (Wichmann et al. 2000). Our data demonstrate that among various carbonaceous UFPs of different chemical composition and surface reactivity, BET surface area (Figure 2C) is most valid in characterizing the efficient particle exposure dose for the lungs. BET surface area appears to be a suitable dose unit, as it integrates number concentration and structural geometrical features of particles such as primary particle size and ultrastructural surface properties. This notion is in agreement with findings reviewed by Oberdorster (1996, 2002), who showed that for fine to ultrafine particles, the particle surface area retained in the alveolar space correlates well with the inflammatory response at higher doses.

A NOAEL of 14 cm^2^ and a LOAEL of 22 cm^2^ BET surface area for mice became evident in our study. Furthermore, the instillation of 16 cm^2^ (2 μg) UfCP particles did not cause a significant inflammatory response, whereas 40 cm^2^ (5 μg) was effective. On the basis of these results, we estimate a threshold of about 20 cm^2^ BET surface area. A threshold of 20 cm^2^ in mice was also suggested by the instillation of relatively high mass doses of quite different particle species by Lison et al. (1997), namely, insoluble manganese dioxide dusts. Printex90 instillation studies in rats indicated a threshold for short-term inflammatory responses of about 130 cm^2^ (Li et al. 1999). Normalizing the particle surface dose to the number of alveolar macrophages or epithelial type I cells (Stone et al. 1992) gives a ratio of 1:8 from mouse to rat; therefore, threshold levels in our study and those in Li’s instillation study are in good agreement. The administration of particles by intratracheal instillation has the advantage of easy delivery of a well-defined dose of the compound of interest to the lungs but have disadvantages that must be mentioned. Instillation causes a preferential deposition in the major conducting airways and results in an uneven distribution pattern within the alveolar region (Leong et al. 1998). Moreover, instillation causes a higher dose rate (particles deposited per time), in contrast to a steadily built-up dose of inhaled particles. Therefore, we performed high-dose inhalation experiments, exposing mice for 24 hr to ufCPs. This resulted in an estimated alveolar dose of at least 3 μg, or 24 cm^2^, which is at the threshold level. The induced effect levels of acute inflammatory end points were slightly elevated compared with clean air exposed controls (e.g., MIP2 = 5.0 (± 0.9) vs. 3.0 (± 0.3) pg/mL, *p* < 0.05) and were within the range between 2- and 5-μg ufCP instillations. Additionally, it is notable that for subchronic particle inhalation in rats, a 200- to 300-cm^2^ threshold level for particle-induced lung effects has been described (Driscoll et al. 1996; Tran et al. 2000). Considering a factor of 8 between mouse and rat, this effect threshold derived from subchronic inhalation is in quite good accordance with the 20-cm^2^ threshold for acute effects derived from our instillation data.

It would certainly be most interesting to extrapolate our experimental findings to man and environmental settings. Converting the experimental threshold of 20 cm^2^ particle surface area from mouse to human, using the same approach described above, results in an estimated critical surface area of about 30,000 cm^2^ for a human. We choose to relate this extrapolated threshold level to particle surface areas encountered at sites of high air pollution, such as busy urban areas with UFP concentrations of up to 10 μg/m^3^. A very rough approximation [assuming UFPs in urban air derived mainly from mobile sources with a specific surface area comparable to DEP (110 m^2^/g), rest ventilating of 15 m^3^/day, and deposition efficiency of 70%] suggests that lung burdens of urban residents may exceed 150 cm^2^/day, which is two orders of magnitude lower than the critical surface dose extrapolated from our data. Individuals suffering from respiratory or cardiovascular diseases might show significant lower effect thresholds and therefore be more susceptible to particulate air pollution. Assuming that deposited particles accumulate in the lungs (Brauer et al. 2001; Semmler et al. 2004), the surface threshold could be reached within months for people living in those areas, but subacute exposure experiments would be more appropriate to assess these scenarios. The threshold level observed here also corresponds only to effects of acute pulmonary inflammation. Other effects, such as cardiovascular consequences, could be more subtle and thus have much lower thresholds. This view is supported by the observation that particle-related cardiovascular effects have been observed in studies where no indication for pulmonary or systemic inflammation could be found (Frampton et al. 2004). Overall, extrapolating findings from experimental studies with model particles to relevant lower environmental concentrations is a very demanding task and must always be undertaken with caution. However, our data contribute to the understanding of UFP toxicity and may be used to support controlled human exposure studies, which must confirm the experimental results.

Epidemiologic studies show no indication of a threshold below which no adverse effects can be found. For example, analysis of data from the 20 largest U.S. cities between 1987 and 1994 provided no evidence for a threshold between PM_10_ and daily cardiorespiratory mortality (Schwartz and Zanobetti 2000; Schwartz et al. 2002). As reviewed by Pope (2000), susceptible individuals, such as the elderly and people with compromised cardiorespiratory function, are more prone to PM burden than healthy individuals. Current discussions suggest that the no-threshold effect in epidemiologic studies cannot be resolved by epidemiologic means in large heterogeneous populations, because some susceptible individuals among the study population are always responding even at very low PM burdens. In our study, age-matched inbred mice with negligible genomic variation and an accurately standardized health status were used. Therefore, our mice may be considered as a specific subpopulation within a typical heterogeneous epidemiologic study population.

In summary, this dose–response assessment of six different types of carbonaceous UFPs confirms the surface area concept within the nanosize range. Therefore, particle surface area may be the most appropriate parameter to evaluate the inflammatory potential and thus predict possible adverse health effects of ultrafine ambient particles. We identified a threshold surface area of approximately 20 cm^2^ for particle exposure in healthy mice, below which no inflammatory response to the instilled particles could be demonstrated in the lung. Despite the well-known limitations of animal-to-human extrapolations, this threshold for acute pulmonary inflammatory reactions might be in relevant proximity to lung burdens of susceptible individuals chronically exposed to highly polluted air.

## Figures and Tables

**Table 1:** Physical and chemical particle characteristics as determined by authors/suppliers.

Particle	Diameter (nm)	OC (%)	Surface area (m^2^/g)
DEP	18–30/–	NA/20	NA/108
PrintexG	30–60/51	1/0.7	43/30
Printex90	12–17/14	2/1	272/300
SootH	8–16/–	19/–	268/–
SootL	8–14/–	7/–	441/–
ufCP	7–12/–	17/–	807/–

Abbreviations: –, not specified; NA, not analyzed. OC was measured according to the NIOSH 5040 method (Cassinelli and O’Connor 1998), and surface area was measured according to the BET method (Brunauer et al. 1938).

**Table 2:** Association between inflammation and particle properties assessed by multiple linear regression analysis.

Effect	Estimate	SE	DF	*t*-Value	Pr > |*t*|
OC	4.43	2.01	156	2.21	0.0286
BET	0.61	0.07	156	9.03	< 0.0001

DF, degrees of freedom; Pr, predictive value (likelihood that there is no association between inflammation and the particle properties OC or BET.
